# Supplementary material for: Distraction decreases rIFG-putamen connectivity during goal-directed effort for food rewards
Source: Sci Rep. 2020 Nov 4;10:19072. doi: 10.1038/s41598-020-76060-y (PMC7643110; doi:10.1038/s41598-020-76060-y)
Supplement: Supplementary file 1 — Supplementary Information. [file 41598_2020_76060_MOESM1_ESM.docx]

*Supplementary Information*

**Distraction decreases rIFG-putamen connectivity during goal-directed effort for food rewards**

**Authors:** Iris Duif, Joost Wegman, Kees de Graaf, Paul A.M. Smeets, Esther Aarts

**Supplementary Methods**

*Liking ratings*

Similar to the analysis of the wanting ratings, we tested whether mean liking ratings for the valued and devalued reward changed significantly over time (using RM-ANOVA with Reward (valued, devalued) and Time (t_0_, t_1_, t_2_, t_3_) as within-subject factors). In addition, we tested for baseline differences (t_0_) in liking of the valued versus devalued reward using a paired-samples t-test.

*Hunger, fullness and thirst ratings*

Hunger, fullness, and thirst ratings were completed 8 times during the experiment (at baseline

(t0), after block 1 and 2 of the task (pre: t1, t2; post: t5, t6), directly before the devaluation (t3),

directly after the devaluation (t4), and after completing the post-devaluation run (t7); Table 2).

Using ANOVA, we tested whether hunger decreased and fullness increased after the devaluation.

Participants were allowed to drink water during the outcome devaluation phase to ensure

participants did not stop eating due to feelings of thirst. Therefore, we did not have specific

hypotheses for changes in thirst.

*Hierarchical regression*

As a secondary analysis, we performed hierarchical regression analyses (stepwise method using backward elimination) to determine effects of individual differences. The effects of distraction on goal-directed effort (button presses) and those on processing of fronto-striatal areas involved in goal-directed control were used as dependent variables. Wanting for the two rewards (valued and devalued, directly before versus directly after the devaluation (t_4_-t_3_)) was added as independent variable to the first level. To the second level, liking of the two rewards (valued and devalued, directly before versus directly after the devaluation (t_4_-t_3_)), performance on the working memory task, hunger, fullness, and thirst ratings (t_4_-t_3_), and the following questionnaires were added: DEBQ, Baratt Impulsiveness Scale (BIS-11 ^1^), Binge Eating Scale (BES ^2^), Power of Food Scale (PFS ^3^), Behavioral Inhibition/Activation System (BIS/BAS ^4^), Kirby monetary choice questionnaire^5^, FFMQ, TFEQ, SFFQ-DHD), see Supplementary Table 1. Note, the DEBQ, BIS-11, BES, PFS, BIS/BAS, and Kirby were filled out on a separate test session prior to the current study.

**Supplementary Results**

*Liking ratings*

Similar to the results of the wanting ratings, the mean liking ratings changed significantly over time for the devalued, but not for the valued, reward (Reward (valued, devalued) x Time (t_0_, t_1_, t_2_, t_3_): *F*(1,35) = 18.13, *p* < 0.001, Table 2). Main effects of Time (*F*(1,35) = 24.54, *p* < 0.001) and Reward (*F*(1,37) = 12.83, *p* = 0.001) were significant, reflecting decreased liking for both rewards after the devaluation, and the devalued reward was generally less liked than the valued reward. At baseline (t_0_), liking did not to differ significantly between the rewards (*t*(1,37) = -2.00, *p* = 0.05).

*Hunger, fullness and thirst ratings*

Analysis of the self-reported hunger, fullness, and thirst ratings showed main effects of Time (see Table 2 for means per time point and *F* and *p* statistics). A paired samples t-test using the mean ratings pre- (t_0_, t_1_, t_2_, t_3_) versus post-devaluation (t_4_, t_5_, t_6_, t_7_), showed participants’ hunger decreased, and their fullness increased significantly after the devaluation (mean (± SEM) hunger pre: 7.0(0.3), mean (± SEM) hunger post: 3.5(0.4), *t*(1,37) = 10.82, *p* < 0.001; mean (± SEM) fullness pre: 1.8(0.2), mean (± SEM) fullness post: 6.1(0.3), *t*(1,37) = -14.91, *p* < 0.001). As anticipated, thirst ratings did not change significantly after the devaluation (*t*(1,37) = .798, *p* < 0.43. Thus, participants were successfully satiated on one of the snacks after the outcome devaluation phase.

*Hierarchical regression*

To determine effects of individual differences, we performed two hierarchical regression (using backward elimination) analyses. We used the effect of distraction on processing of the rIFG, and on connectivity between rIFG and left putamen during goal-directed control as dependent variables (interaction effect of Load x Reward x Time for the rIFG, and the psychophysiological interaction effect of Load x Reward x Time for left putamen with the rIFG seed). The independent variables were: wanting (first level), liking, performance, hunger, fullness, and thirst ratings, and the questionnaires (second level) as described in the *Methods* section. The significance level was Bonferroni-corrected for the two outcome measures and set to α = 0.025. Both regression analyses did not result in significant models (all *p* > 0.025). Thus, there were no individual factors predicting the effect of distraction on goal-directed control in the rIFG or distraction-related connectivity between the rIFG (seed) and left putamen.

**Supplementary Tables**

**Supplementary Table S1.** Neuropsychological measurements

|  | Mean | Standard deviation | Minimum | Maximum |
| --- | --- | --- | --- | --- |
| BIS | 15.8 | 3.1 | 10 | 24 |
| BAS | 24.6 | 5.1 | 18 | 35 |
| BIS-11 | 69.1 | 5.3 | 59 | 80 |
| Kirby | 0.0070 | 0.0080 | 0.0002 | 0.0255 |
| BES | 24 | 4.8 | 16 | 33 |
| SFFQ-DHD | 54 | 13.0 | 24 | 75 |
| DEBQ |  |  |  |  |
| *Restraint* | 2.2 | 0.7 | 1.0 | 3.9 |
| *Emotional* | 2.2 | 0.6 | 1.2 | 3.4 |
| *External* | 3.3 | 0.5 | 2.6 | 4.3 |
| PFS | 35.3 | 9.3 |  |  |
| TFEQ |  |  |  |  |
| *Diet* | 7.2 | 3.7 | 1.0 | 18.0 |
| *Disinhibition* | 5.2 | 2.7 | 2.0 | 14.0 |
| *Hunger* | 6.1 | 2.9 | 0.0 | 12.0 |
| FFMQ | 81.9 | 9.0 | 60.0 | 101.0 |

*BIS/BAS:* Behavioural Inhibition System/Behavioral Approach System questionnaire; *BIS-11*: Baratt Impulsiveness Scale-11; *Kirby*: delayed reward discounting questionnaire; *BES*: Binge Eating Scale; *SFFQ-DHD*: Short Food Frequency Questionnaire, Dutch Healthy Diet; *DEBQ*: Dutch Eating Behaviour Questionnaire; *PFS:* Power of Food Scale; *TFEQ*: Three Factor Eating Questionnaire; FFMQ: Five Facet Mindfulness Questionnaire. Note, the DEBQ, BIS-11, BES, PFS, BIS/BAS, and Kirby were filled out on a separate test session prior to the current study. N=38.

**Supplementary Table S2.** Self-reported hunger, fullness, and thirst ratings, and wanting and liking ratings for each food reward (valued, devalued).

|  | t _(0)_ | t _(1)_ | t _(2)_ | t _(3)_ | t _(4)_ | t _(5)_ | t _(6)_ | t _(7)_ | *p* | *F* |
| --- | --- | --- | --- | --- | --- | --- | --- | --- | --- | --- |
| *Hunger, fullness, and thirst ratings* | | | | | | | | | | |
| Hunger | 7.2(0.3) | 7.5(0.3) | 7.3(0.4) | 6.1(0.4) | 2.8(0.4) | 3.8(0.4) | 3.7(0.4) | 3.6(0.4) | <0.001 | 24.20 |
| Fullness | 1.4(0.2) | 1.5(0.2) | 1.5(0.2) | 3.0(0.3) | 7.1(0.3) | 5.9(0.4) | 5.9(0.4) | 5.6(0.4) | <0.001 | 68.98 |
| Thirst | 4.6(0.4) | 5.8(0.4) | 6.2(0.4) | 4.0(0.4) | 5.0(0.3) | 5.0(0.3) | 5.0(0.3) | 4.7(0.3) | <0.001 | 6.17 |
| *Wanting for each reward* | | | | | | | | | | |
| Valued | 6.7(0.4) | - | - | 6.4(0.4) | 5.9(0.4) | - | - | 6.3(0.4) | 0.40 | 1.01 |
| Devalued | 6.0(0.5) | - | - | 6.7(0.4) | 1.8(0.3) | - | - | 2.9(0.3) | <0.001 | 41.00 |
| *Liking for each reward* | | | | | | | | | | |
| Valued | 7.4(0.3) | - | - | 7.4(0.3) | 6.9(0.3) | - | - | 7.2(0.3) | 0.26 | 1.41 |
| Devalued | 8.2(0.2) | - | - | 7.6(0.3) | 4.4(0.4) | - | - | 5.1(0.3) | <0.001 | 38.29 |

Means and standard errors per time point, and Time statistics are shown.

**Supplementary Table S3.** Snack intake

|  | Intake | | n | |
| --- | --- | --- | --- | --- |
|  | Amount (grams; mean ± SD) | Energy (kilojoules; mean ± SD) | |  |
| Sweet snack | 98.1 ± 40.5 | 1905.8 ± 828.4 | 19 | |
| M&Ms | 97.9 ± 42.4 | 1966.4 ± 852.4 | 17 | |
| Skittles | - | - | 0 | |
| Wine gums | 99.5 ± 26.2 | 1390.5 ± 365.7 | 2 | |
|  |  |  |  | |
| Salty snack | 93.7 ± 44.7 | 2108.0 ± 1082.2 | 18 | |
| Pringles (original) | 99.8 ± 34.3 | 1500.5 ± 775.0 | 11 | |
| Cocktail nuts (crusted peanuts) | 137.5 ± 108.2 | 3325.2 ± 2607.9 | 2 | |
| Salty crackers | 62.8 ± 18.8 | 1300.6 ± 388.9 | 5 | |

Means and standard deviations (SD) are presented per snack type (across the sweet and salty snacks and per snack) for the total amount (grams) and energy (kilojoules) participants consumed during the outcome devaluation phase (fixed portion + *ad libitum* amount). Note that n=37, as these data were not available for one subject due to technical problems. As none of the participants consumed Skittles during the devaluation phase, these cells contain no values. Snack devaluation was counter-balanced, i.e. participants were devalued on only one of the snacks (sweet or salty snack).

**Supplementary Figures**

**

**

**Supplementary Figure S1**. Timeline of the experimental session. After the preparations, participants performed the distraction + effort task in the MR-scanner twice, i.e., before and after outcome devaluation of one of the snacks (sweet or salty) through satiation. Pre- and post-devaluation, participants performed 100 trials (4 blocks of 25 trials). To manipulate distraction, 50% of trials were of low load and 50% of high load. To measure shifts in goal-directed behavior, the low and high load conditions had 25 trials in which the sweet reward could be won, and 25 trials in which the salty reward could be won. After the task (pre- and post-devaluation), participants consumed 1/5th of their sweet and salty winnings outside the MR scanner. VAS hunger, fullness, thirst (HFT), wanting and liking (after tasting) were rated at several time points. See the Methods section for further details. VAS = visual analogue scale; HFT = hunger, fullness, thirst; S-O = stimulus-outcome.

**
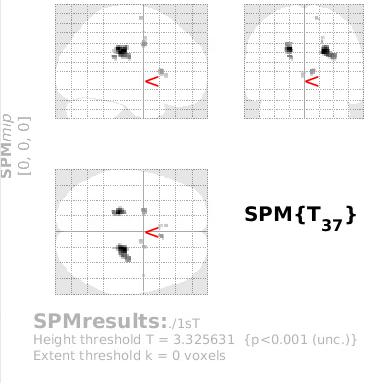
**

**
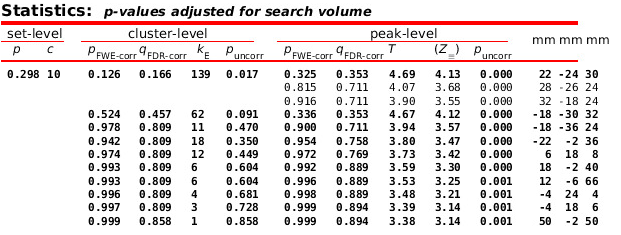

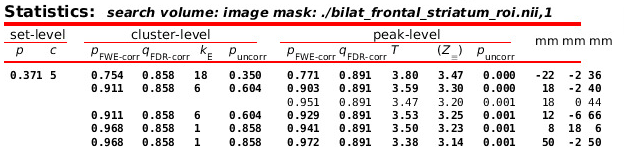
**

**Supplementary Figure S2.** vmPFC gPPI results: Reward(**val>dev**)*Time(post>pre)*Load(low>high)

**
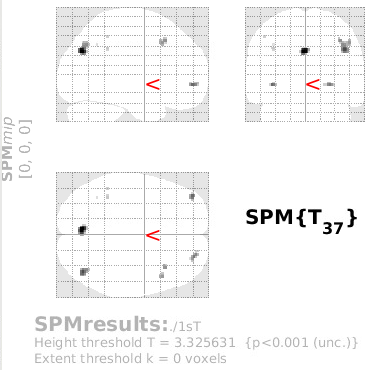
**

**
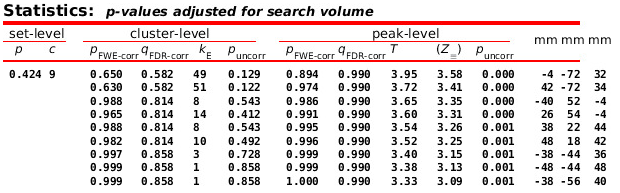
**

**
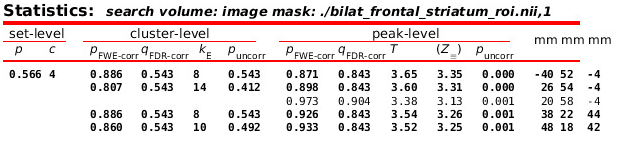
**

**Supplementary Figure S3.** vmPFC gPPI results: Reward(**dev>val**)*Time(post>pre)*Load(low>high)

**Supplementary References**

1. Patton, J. H., Stanford, M. S. & Barratt, E. S. Factor structure of the barratt impulsiveness scale. *J. Clin. Psychol.* **51**, 768–774 (1995).

2. Gormally, J., Black, S., Daston, S. & Rardin, D. The assessment of binge eating severity among obese persons. *Addict. Behav.* **7**, 47–55 (1982).

3. Lowe, M. R. *et al.* The Power of Food Scale. A new measure of the psychological influence of the food environment. *Appetite* **53**, 114–118 (2009).

4. Carver, C. S. & White, T. L. Behavioral inhibition, behavioral activation, and affective responses to impending reward and punishment: The BIS/BAS Scales. *J. Pers. Soc. Psychol.* **67**, 319–333 (1994).

5. Kirby, K. N. One-year temporal stability of delay-discount rates. *Psychon. Bull. Rev.* **16**, 457–462 (2009).
